# Objective predictors of intern performance

**DOI:** 10.1186/s12909-021-02487-0

**Published:** 2021-01-26

**Authors:** Amanda C. Filiberto, Lou Ann Cooper, Tyler J. Loftus, Sonja S. Samant, George A. Sarosi, Sanda A. Tan

**Affiliations:** 1 Department of Surgery, University of Florida Health, 1600 SW Archer Ave, PO Box 100109, Gainesville, FL 32610 USA; 2 Office for Educational Affairs, University of Florida College of Medicine, Gainesville, FL USA; 3 University of Florida College of Medicine, Gainesville, FL USA

**Keywords:** Medical student, Internship, Performance, Residency, Medical education

## Abstract

**Background:**

Residency programs select medical students for interviews and employment using metrics such as the United States Medical Licensing Examination (USMLE) scores, grade-point average (GPA), and class rank/quartile. It is unclear whether these metrics predict performance as an intern. This study tested the hypothesis that performance on these metrics would predict intern performance.

**Methods:**

This single institution, retrospective cohort analysis included 244 graduates from four classes (2015–2018) who completed an Accreditation Council for Graduate Medical Education (ACGME) certified internship and were evaluated by program directors (PDs) at the end of the year. PDs provided a global assessment rating and ratings addressing ACGME competencies (response rate = 47%) with five response options: excellent = 5, very good = 4, acceptable = 3, marginal = 2, unacceptable = 1. PDs also classified interns as outstanding = 4, above average = 3, average = 2, and below average = 1 relative to other interns from the same residency program. Mean USMLE scores (Step 1 and Step 2CK), third-year GPA, class rank, and core competency ratings were compared using Welch’s ANOVA and follow-up pairwise t-tests.

**Results:**

Better performance on PD evaluations at the end of intern year was associated with higher USMLE Step 1 (*p* = 0.006), Step 2CK (*p* = 0.030), medical school GPA (*p* = 0.020) and class rank (*p* = 0.016). Interns rated as average had lower USMLE scores, GPA, and class rank than those rated as above average or outstanding; there were no significant differences between above average and outstanding interns. Higher rating in each of the ACGME core competencies was associated with better intern performance (*p* < 0.01).

**Conclusions:**

Better performance as an intern was associated with higher USMLE scores, medical school GPA and class rank. When USMLE Step 1 reporting changes from numeric scores to pass/fail, residency programs can use other metrics to select medical students for interviews and employment.

**Supplementary Information:**

The online version contains supplementary material available at 10.1186/s12909-021-02487-0.

## Introduction

In selecting medical students for interview and employment, residency programs seek candidates that will perform well as residents and become excellent physicians. The definition of a successful intern likely varies by specialty, but an individual’s work competence, organizational acumen, social intelligence and personal characteristics are major contributors [1]. The Accreditation Council for Graduate Medical Education (ACGME) uses competency based medical education and specialty specific Milestones as a systematic framework to evaluate resident performance and determine promotion to the next level of training [2]. Selection committees consider a variety of factors when offering interviews and creating rank lists, including but not limited to standardized test scores, class rank, clerkship performance, letters of recommendation, and personal statements [3–5]. Determining which objective measures are most predictive of residency performance could help guide selection committees and program directors (PDs).

Historically, residency programs have relied on United States Medical Licensing Examination (USMLE) scores as an objective and consistent measure of medical student performance across different medical schools [4]. Among the 2018 National Resident Matching Program (NRMP) survey of program directors, 94% of the ~ 1200 programs cited USMLE Step 1 score as an important factor for selecting candidates to interview, and more than half reported the use of a prescribed cutoff score, below which applicants are ineligible [6]. In addition to their known use as a screening tool, USMLE scores have been shown to correlate with the NRMP rank list [7] as well as with in service-training exams [4, 5, 8]. However, USMLE scores have had inconsistent relationships with intern performance as assessed by faculty evaluations for several medical specialties [3–5, 8–10]. Additionally, on February 12, 2020 the USMLE announced that Step 1 results will now be reported as a pass/fail outcome, as compared with the previously reported 3-digit numerical score [11]. Therefore, other objective predictors of intern performance may become increasingly important in the process of selecting medical students for interview and employment.

Few studies have evaluated medical school metrics and intern performance across all specialties, and most of the existing studies are small, single institution and involve a single specialty, and thus are limited in generalizability [12, 13]. Due to the lack of consensus regarding the strength and validity of different applicant characteristics as predictors of residency performance, we collected objective data on graduating medical students from a single institution and obtained standardized evaluations from their respective PDs as a metric of intern performance. The aim of this study was to assess independent predictors of intern performance, so that these predictors can be used to accurately and precisely rank future applicants. This study tested the hypothesis that performance on USMLE scores, grade-point average (GPA), class rank, and class quartile would predict intern performance.

## Methods

This single-institution retrospective cohort analysis included 244 graduates from the University of Florida College of Medicine during a four-year period (2015–2018) who subsequently completed an ACGME certified internship. Institutional Review Board approval was obtained. To assess intern performance, standardized evaluation forms were provided to residency PDs with a signed authorization form (Appendix A) at the conclusion of each year, surveyed annually for 4 years. The evaluation form was provided to PDs at the conclusion of the resident’s first year, and PDs were instructed to evaluate the resident as a first year intern. PDs provided a global assessment rating and ratings on specific items addressing the six ACGME core competencies (response rate = 47%). Evaluation items had five response options: excellent = 5, very good = 4, acceptable = 3, marginal = 2, and unacceptable = 1. PDs were also asked to classify interns as outstanding (*n* = 85), above average (*n* = 95), average (*n* = 60), or below average (*n* = 4) relative to the other interns in their cohort (cohort comparison rating).

**Fig. 1:**
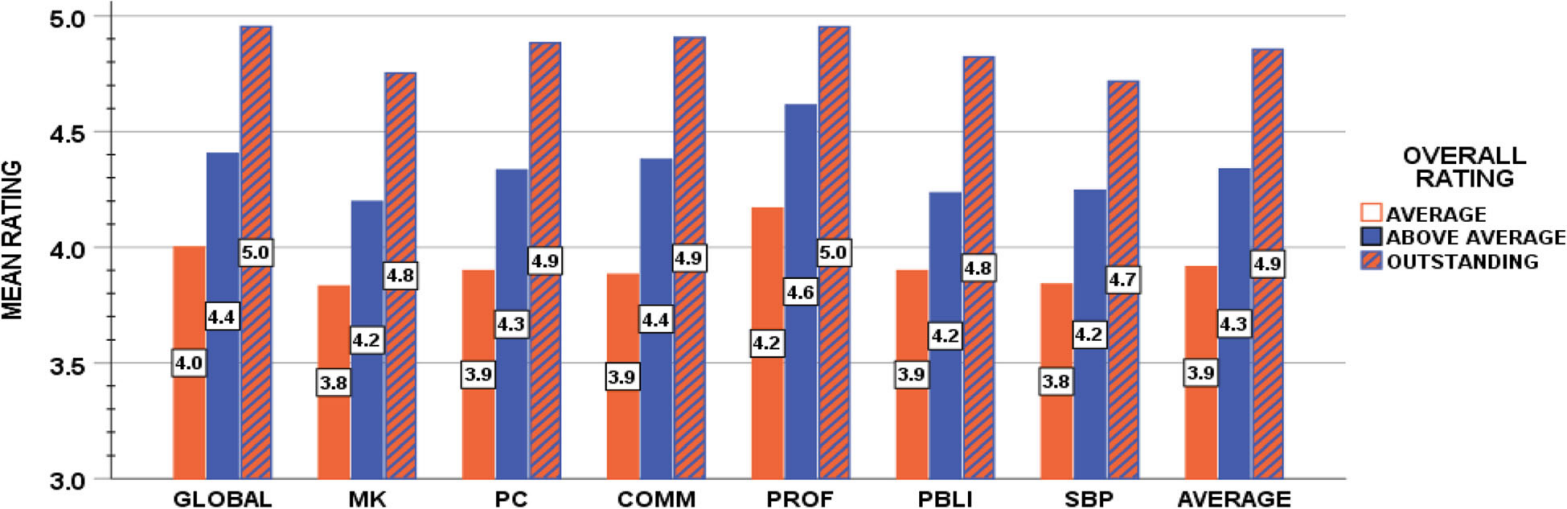
Mean for evaluation items by overall rating by PDs for graduates after intern year. MK, medical knowledge; PC, patient care; Comm, communication skills; Prof, professionalism; PBLI, practice based learning and improvement; SBP, systems based practice

The medical school curriculum at our institution has had no significant changes during this period of time. Means for these metrics were compared between graduating classes, with subgroup analyses of interns who were evaluated by PDs and interns that were not evaluated by PDs. These analyses found no statistical differences (*p* < 0.05). We also found similar distributions of specialties in the two groups (Table 1); therefore, the data for all 4 years were analyzed in aggregate. Descriptive statistics and analyses were calculated using SAS software version 9.4 (SAS Institute Inc., Cary, NC); Fig. 1 was constructed using SPSS version 25 (IBM Corp., Armonk, N.Y). Mean USMLE scores (Step 1 and Step 2CK), third-year GPA (required clinical clerkships), class rank, and core competency ratings were compared using Welch’s ANOVA and follow-up pairwise t-tests.

**Table 1:** Medical school graduates by specialty

	All graduates	Graduates with PD evaluations
**Specialty, n (%)**		
Anesthesia	33 (6)	23 (9)
Dermatology	15 (3)	6 (2)
Diagnostic radiology	20 (4)	10 (4)
Emergency medicine	64 (12)	31 (13)
Family medicine	32 (6)	17 (7)
General surgery	30 (6)	8 (3)
IM/Peds	3 (1)	1 (0)
Internal medicine	99 (19)	46 (19)
IR	1 (0)	1 (0)
Neurology	8 (2)	4 (2)
Neurosurgery	11 (2)	1 (0)
OB/Gyn	47 (9)	25 (10)
OMFS	14 (3)	2 (1)
Ophthalmology	14 (3)	5 (2)
Orthopedic	16 (3)	5 (2)
Otolaryngology	6 (1)	2 (1)
Pathology	6 (1)	1 (0)
Pediatrics	58 (11)	35 (14)
Plastic surgery	3 (1)	1 (0)
PM&R	2 (0)	1 (0)
Psychiatry	19 (4)	12 (5)
Radiation oncology	9 (2)	4 (2)
Urology	9 (2)	4 (2)
Vascular surgery	0 (0)	0 (0)
Thoracic surgery	2 (0)	0 (0)

*PD* program director, *IM* internal medicine, *IR* interventional radiology, *Peds* pediatrics, *OB/Gyn* obstetrics and gynecology, *OMFS* oral maxillofacial surgery, *PM&R* physical medicine and rehabilitation

## Results

There were a total of 521 graduates from the University of Florida College of Medicine from 2015 to 2018. Two hundred and forty-four graduates were evaluated by their PDs using a standardized evaluation form (response rate = 47%). Interns in the following specialties had the highest PD response rates: internal medicine (17%), emergency medicine (14%), pediatrics (14%), and obstetrics and gynecology (10%). There were no significant differences among graduates that received evaluations and those that did not (Table 1). Six percent of all University of Florida graduates matriculated into general surgery, which is similar to the 2020 national average of 4.7%. Subgroup analyses demonstrated that students matching in general surgery and other surgical specialties had baseline characteristics that were similar to students matching into non-surgical specialties. There were no significant differences among the four classes of medical student graduates (2015–2018) in terms of performance metrics, core competencies or global assessment.

Descriptive statistics of the sample stratified by PD’s assessment of intern performance (relative to other interns from the same residency program) are shown in Table 2. Descriptive statistics are provided for the students evaluated as below average (*n* = 4), but this group is not included in the statistical analyses. One-way ANOVA showed higher GPA (*p* = 0.02) and class rank (*p* = 0.016) were associated with better performance, as indicated by PD overall assessment of intern performance as average, above average or outstanding. Better intern performance was also associated with higher USMLE Step 1 (*p* = 0.006) and Step 2CK (*p* = 0.030) scores. Pairwise t-tests demonstrated that interns rated as average had lower scores, lower GPA, and were more likely to have a lower class rank than interns rated as above average or outstanding. Although on average the metrics were higher for interns rated outstanding than for those rated above average, these differences were not statistically significant.

**Table 2:** Medical school performance metrics stratified by program director’s overall assessment of intern performance (relative to their cohort) at the end of intern year

	Below Average^a^	Average	Above Average	Outstanding			Follow up t-tests for significant ANOVA					
	(*n* = 4)	(*n* = 60)	(*n* = 95)	(*n* = 85)	ANOVA		A vs. AA		A vs. O		AA vs. O	
Performance metrics	M (SD)	M (SD)	M (SD)	M (SD)	*F*	*p*	*t*	*p*	*t*	*p*	*t*	*p*
USMLE Step 1	210.5 (24.0)	224.6 (19.3)	232.4 (18.6)	234.6 (17.4)	5.3	< 0.01	2.5	0.01	3.2	< 0.01	0.9	0.38
USMLE Step 2CK	241.5 (15.8)	243.4 (16.1)	249.1 (15.9)	250.4 (15.6)	3.6	0.03	2.1	0.04	2.6	< 0.01	0.6	0.54
MS3 GPA	3.2 (0.1)	3.4 (0.4)	3.5 (0.3)	3.6 (0.3)	4.0	0.02	2.4	0.02	3.0	< 0.01	0.7	0.48
MS3 Rank	105.8 (11.6)	76.8 (37.5)	63.1 (38.6)	58.5 (38.8)	4.2	0.02	−2.1	0.04	−2.8	< 0.01	−0.8	0.41

^a^Descriptive statistics provided for the students evaluated as “below average”, but this group is not included in the statistical analyses reported in this table

Figure 1 shows the results of the mean scores for the evaluation items using the overall rating as the grouping variable. Higher rating in each of the core competencies was associated with better performance as an intern, as indicated by PD overall assessment of intern performance as average, above average or outstanding. For global assessment, the items based on ACGME core competencies and the average of these seven items, the clear differences shown in the graph are significant. Pairwise t-tests demonstrated that interns rated as average had lower competency ratings than those rated as above average or outstanding (*p* < 0.01). A chi square test of association between the global assessment rating and the overall cohort comparison rating demonstrated a strong relationship between these two measures, χ2 (9) = 299.4, *p* < 0.0001.

## Discussion

Intern performance evaluations were significantly associated with objective measures of academic performance in medical school. These findings were consistent when interns were compared to their peers as well as a global assessment by PDs. There were no significant differences between above average and outstanding interns, reflecting the variability and overlap of the distribution of performance metrics among graduates that are making successful progress in their training.

With the introduction of policy changes to USMLE score reporting, it is important to consider other metrics that can be used by PDs to evaluate the growing pool of residency applicants. In addition to Step 1, our study found a significant association between performance as an intern and GPA, class rank and Step 2 CK. As the Step 2 CK examination places greater emphasis on the clinical application of medical knowledge, it may represent a better predictive measure of clinical performance. Unlike the USMLE, preclinical coursework and grading schemes vary substantially from school to school, which make it difficult to compare applicants but may be a useful tool for PDs evaluating multiple applicants from a single institution. Quartile designation, based on class rank determined by third year GPA, is included in the MSPE (Dean’s letter) and may offer a way to compare applicants.

As the number of applicants to training programs continues to climb while the number of positions available remains unchanged [14], residency programs continue to face the difficult task of selecting future residents. The level of scrutiny of academic variables is difficult to quantify as residency programs typically do not publish criteria used to interview or score applicants. USMLE scores appear to be related to success in the match process [15], as specialties with the highest percentages of unmatched U.S. seniors such as otolaryngology and neurosurgery [14] have higher USMLE Step 1 and Step 2 CK scores [16]. Further, several studies have shown an association between USMLE scores and performance on in service-training examinations across several specialties [17–20]. However, few studies have evaluated which factors predict success as a resident.

The results of our study are consistent with findings from several other studies regarding both overall performance as an intern and academic measures in medical school. In a study of 338 medical student graduates from a single institution, Alexander et al. [13] found that higher GPA, and USMLE Step 1 and Step 2 were each associated with better PD assessments of overall performance as an intern. Paolo et al. [12] found similar results when surveying PDs of 382 interns, reporting that residents with higher GPA and USMLE Step 1 and Step 2 scores in medical school were rated higher than those with lower scores. A study by Andriole et al. [9] that involved 87 recent graduates from a single medical school who pursued surgical training found that Step 2 was the only significant predictor of intern performance.

**I**n contrast, several specialty-specific studies have reported that medical school metrics are not significantly associated with resident performance. Fryer et al. were unable to consistently predict any type of obective metric with general surgery resident performance [21]. A small retrospective study of 57 neurology residents by Burish et al. [3] found that Step 1 scores did not correlate with overall neurology resident quality, although it may predict success on future standardized medical examinations. In a study of 69 pediatric house officers, Borowitz et al. [8] found that neither medical school grades or performance on standardized exams were predictors of clinical performance during pediatric residency. Discrepancies in previously reported results may be attributable to the smaller sample sizes in the specialty-specific studies, rendering them underpowered to detect significant associations between medical school metrics and resident performance.

The single-institution, retrospective design of this study limits its generalizability. The analyses performed in this study, however, have not been previously reported using national data, and the authors believe that findings from this study may be useful to educators and serve as framework for larger, multi-center studies. While our overall survey response rates were much higher than the NRMP PD survey response rates [6], data were missing for approximately 53% of the overall cohort. Further, among the known limitations associated with survey response bias, there is the possibility of the halo effect, where raters tend to rely on general perceptions even when they are asked to evaluate specific characteristics of individuals [22]. Evaluators may also exhbit central tendency, where ratings are limited to values near the midpoint of the rating scale, avoiding extreme ratings [23]. Grade inflation is also a well-recognized limitation in education literature. Evaluators across specialties may interpret survey questions differently, leading to inconsistencies and response bias. In addition to a global assessment, interns were assessed by their PDs relative to their peers, introducing the possibility that evaluations were confounded by differences in the quality of interns in different specialties and at different hospitals. Ranking systems such as the *U.S. News & World Report* lack objective quality measurements [24], and do not provide data for all program specialty and subspecialties, and therefore were not included in our analysis.

Although our study found associations between both Step 1 and Step 2 CK and intern performance, with the introduction of the new score reporting system for Step 1, more of an emphasis may be placed on additional metrics such as GPA, class rank and Step 2 CK scores in the future and should be further evaluated. Additionally, although objective measures play a significant role in the evaluation of applicants for residency positions, the importance of subjective factors, such as baseline personal characteristics should be emphasized [25].

## Conclusions

Better performance as an intern was associated with higher USMLE scores, medical school GPA and class rank. When USMLE Step 1 reporting changes from numeric scores to pass/fail, residency programs can use other metrics to predict clinical performance. Although no single factor can be used to accurately predict performance in residency, these findings provide a framework for using objective metrics to select medical students for interview and employment, seeking candidates that will perform well as residents and become excellent physicians.

## Supplementary Information

**Additional file 1.** Appendix A: Program Director Evaluator Form.

## Data Availability

The datasets used and/or analyzed during the current study are available from the corresponding author on reasonable request.

## Abbreviations

USMLE: United States Medical Licensing Examination
GPA: Grade-point average
ACGME: Accreditation Council for Graduate Medical Education
PDs: Program directors
