# Supplementary material for: Objective predictors of intern performance
Source: BMC Med Educ. 2021 Jan 26;21:77. doi: 10.1186/s12909-021-02487-0 (PMC7839184; doi:10.1186/s12909-021-02487-0)
Supplement: Supplementary file 1 — Additional file 1. Appendix A: Program Director Evaluator Form. [file 12909_2021_2487_MOESM1_ESM.docx]

**Appendix A: Program Director Evaluator Form**

Thank you in advance for completing this survey. Your assessments of how well the University of Florida prepared our graduates for your residency are important for our ongoing review of our educational program and in accordance with LCME requirements.

** Please Enter PG Name:***

**
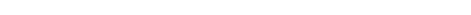
**

** Global Assessment***

o Excellent

o Very Good

o Acceptable

o Marginal

o Unacceptable

o Cannot Evaluate

** Medical Knowledge- Basic and Clinical Science***

o Excellent

o Very Good

o Acceptable

o Marginal

o Unacceptable

o Cannot Evaluate

** Patient Care - Clinical Judgment, Patient Management, Diagnostic Skills***

o Excellent

o Very Good

o Acceptable

o Marginal

o Unacceptable

o Cannot Evaluate 

**Technical/ Surgical Skills***

o Excellent

o Very Good

o Acceptable

o Marginal

o Unacceptable

o Cannot Evaluate

** Communication Skills - Oral and Written***

o Excellent

o Very Good

o Acceptable

o Marginal

o Unacceptable

o Cannot Evaluate

** Professional Conduct/ Behavior***

o Excellent

o Very Good

o Acceptable

o Marginal

o Unacceptable

o Cannot Evaluate

** Practice - Based Learning and Improvement***

o Excellent

o Very Good

o Acceptable

o Marginal

o Unacceptable

o Cannot Evaluate

** Systems - Based Practice***

o Excellent

o Very Good

o Acceptable

o Marginal

o Unacceptable

o Cannot Evaluate

** How many PGY-1 UF residents are in your program?***

o1

o2

o3

o4

o 5 or more

** Compared to other residents in your program, how would you rank UF PGY-1 graduates?***

o Outstanding

o Above Average

o Average

o Below Average
